# TWEAK/Fn14 mediates atrial‐derived HL‐1 myocytes hypertrophy via JAK2/STAT3 signalling pathway

**DOI:** 10.1111/jcmm.13724

**Published:** 2018-07-04

**Authors:** Li Hao, Manyi Ren, Bing Rong, Fei Xie, Ming‐jie Lin, Ya‐chao Zhao, Xin Yue, Wen‐qiang Han, Jing‐quan Zhong

**Affiliations:** ^1^ The Key Laboratory of Cardiovascular Remodeling and Function Research Chinese Ministry of Education Chinese Ministry of Health Qilu Hospital Shandong University Jinan China; ^2^ Department of Cardiology Shandong Provincial Qianfoshan Hospital Shandong University Jinan China

**Keywords:** Fn14, HL‐1 atrial myocytes, hypertrophy, JAK2/STAT3, TWEAK

## Abstract

Atrial myocyte hypertrophy is one of the most important substrates in the development of atrial fibrillation (AF). The TWEAK/Fn14 axis is a positive regulator of cardiac hypertrophy in cardiomyopathy. This study therefore investigated the effects of Fn14 on atrial hypertrophy and underlying cellular mechanisms using HL‐1 atrial myocytes. In patients with AF, Fn14 protein levels were higher in atrial myocytes from atrial appendages, and expression of TWEAK was increased in peripheral blood mononuclear cells, while TWEAK serum levels were decreased. In vitro, Fn14 expression was up‐regulated in response to TWEAK treatment in HL‐1 atrial myocytes. TWEAK increased the expression of ANP and Troponin T, and Fn14 knockdown counteracted the effect. Inhibition of JAK2, STAT3 by specific siRNA attenuated TWEAK‐induced HL‐1 atrial myocytes hypertrophy. In conclusion, TWEAK/Fn14 axis mediates HL‐1 atrial myocytes hypertrophy partly through activation of the JAK2/STAT3 pathway.

## 1 INTRODUCTION

Atrial fibrillation (AF) is the most common sustained arrhythmia in clinical practice, and its underlying substrates are the focus of research on novel therapeutic targets.^1^ Atrial myocyte hypertrophy is one of the most important structural remodelling features in AF and its main substrate.^2^ However, the molecular mechanisms underlying atrial myocyte hypertrophy are largely unknown.

Tumour necrosis factor (TNF)‐like weak inducer of apoptosis (TWEAK) is a multifunctional cytokine that functions mainly through the fibroblast growth factor‐inducible molecule 14 (Fn14) receptor.^3^ Fn14 is a tightly regulated receptor that has been associated with various downstream signalling cascades.^4^, ^5^ Although Fn14 usually is expressed at very low levels under physiological conditions,^6^ it can be induced in cardiovascular disease,^7^, ^8^ and the TWEAK/Fn14 axis is involved in the pathogenesis of various cardiovascular diseases.^4^, ^5^, ^6^, ^7^, ^8^ Adult TWEAK transgenic mice exhibit cardiomyocyte hypertrophy and cardiac dilation, while Fn14‐/‐ mice are protected against TWEAK‐induced cardiac dilation.^6^ Activation of TWEAK/Fn14 signalling in adult rat primary cardiomyocytes causes cardiomyocyte hypertrophy.^9^ However, atrial expression of Fn14 in patients with AF and its role in atrial structural remodelling remains undefined.

Janus kinase (JAK)/signal transducer and activator of transcription (STAT) pathway was initially discovered as a major cytokine signal transduction pathway.^10^ JAK‐STAT signalling pathway plays an important role in cardiac pathophysiology and has been implicated in pressure overload‐induced cardiac hypertrophy and remodelling.^11^ The activation of JAK/STAT pathway by IL‐6 may contribute to the pathogenesis of cardiac hypertrophy,^12^ and phospho‐STAT3 levels are elevated in atrial tissues from AF patients.^1^ TWEAK/Fn14 up‐regulates pro‐inflammatory cytokine secretion via STAT3 pathways in hepatic stellate cells.^13^

We hypothesized that up‐regulated Fn14 expression may facilitate TWEAK‐induced atrial myocyte hypertrophy and that, conversely, Fn14 inhibition may have a protective role on atrial structural remodelling. This study therefore explored the potential role and underlying mechanism of Fn14 in HL‐1 atrial myocyte hypertrophy under conditions of TWEAK stimulation.

## 2 MATERIALS AND METHODS

### 2.1 Cell culture and transient transfection

HL‐1 atrial myocytes derived from adult mouse atria were obtained from Dr. William Claycomb (Louisiana State University, New Orleans, LA, USA) and cultured in the Claycomb medium as previously described.^14^, ^15^ HL‐1 myocytes were cultured in antibiotic‐free growth medium until they had reached 60% confluency. The cells were incubated with a mixture of specific small interfering RNA (siRNA) to Fn14, JAK2 or STAT3 (GenePharma, Shanghai, China) and Lipo‐fectamine 2000 (Invitrogen, CA, USA) in antibiotic‐free and serum‐free medium for 12 hours. Control cells were treated with siRNA negative control. The target sequence for Fn14 siRNA was as follows: sense, 5′‐GCUGGUUUCUAGUUUCCUGTT‐3′, antisense, 5′‐CAGGAAACUAGAAACCAGCTT‐3′; and for negative control (NC): sense, 5′‐UUCUCCGAACGUGUCACGUTT‐3′, antisense, 5′‐ACGUGACACGUUCGGAGAATT ‐3′. The siRNA sequence for JAK2 was: sense, 5′‐GCUCAAAUGAAAGUAGAAUTT‐3′, antisense, 5′‐AUUCUACUUUCAUUUGAGCTT‐3′; and for STAT3: sense, 5′‐GGGUCUCGGAAAUUUAACATT‐3′, antisense, 5′‐UGUUAAAUUUCCGAGACCCTT‐3′. After transfection, cells were incubated in growth medium for 24 hours and then stimulated. Recombinant mouse TWEAK was purchased from R&D Systems (Minneapolis, MN, USA) and reconstituted with sterile phosphate‐buffered saline (PBS) to 100 μg/mL and stored at −20°C.

### 2.2 Isolation of PBMCs from blood samples

Peripheral blood samples obtained by venipuncture from patients with AF or from controls with normal sinus rhythm (NSR) were collected in tubes treated with EDTA‐K2. Exclusion criteria included: (*i*) Severe cardiomyopathy, valvular disease, congenital heart disease, decompensated heart failure and left ventricular dysfunction; (*ii*) malignant tumours, systemic inflammatory disease, hyperthyroidism, connective tissue disease, liver and kidney dysfunction; (*iii*) history of acute cardiovascular and cerebrovascular events, trauma or surgery within three previous months; (*iv*) on immunosuppressive therapy or anti‐inflammation therapy; (*v*) reversible atrial fibrillation caused by thyroid hyperfunction, alcohol drinking, surgery or other causes. Peripheral blood mononuclear cells (PBMCs) then were separated using lymphocyte separation medium (TBDsciences, Tianjin, China) following manufacturer's protocol.

### 2.3 Western blotting

Total protein was extracted from HL‐1 cells or PBMCs using standard protocols in a RIPA buffer with 1 mmol/L PMSF. Equal amounts of protein from different experimental groups were separated by 8%‐12% SDS‐PAGE and transferred to PVDF membranes (Millipore, Eschborn, Germany), which were blocked for 1 hour with 5% non‐fat milk in TBST at room temperature, then incubated overnight at 4°C with primary antibodies against TWEAK (Abcam, Cambridge, UK), Fn14 (Abcam, Cambridge, UK), ANP (Millipore, Temecula, CA), Troponin T (Novus, Littleton, CO, USA), β‐actin (Millipore), phospho‐JAK1 (at Tyr1022/1023), JAK1, phospho‐JAK2 (at Tyr1007/1008), JAK2, phospho‐TYK2 (at Tyr1054/1055), TYK2, phospho‐STAT1 (at Tyr701), STAT1, phospho‐STAT3 (at Tyr705), and STAT3 (all Cell Signaling Technology, Danvers, MA), and then incubated with appropriate secondary HRP‐conjugated secondary antibody for 1 hour at room temperature. The protein signals were detected using a chemiluminescence kit (Millipore, MA, USA).

### 2.4 Histology and immunohistochemistry

Human atrial appendages were dissected and fixed immediately in 4% paraformaldehyde. Tissue was paraffin‐embedded and sectioned (5 μm) for subsequent analyses. Atrial myocyte area (μm^2^) was measured in images from H&E‐stained sections from human atrial appendages. Immunohistochemistry was performed for detecting Fn14 expression, and sections were incubated overnight at 4°C with primary antibodies against Fn14 (1:100 dilution; OmnimAbs, Alhambra, CA, USA). Goat anti‐rabbit antibody was used as secondary antibody. All the results were analysed by Image‐Pro Plus 6.0 (Media Cybernatics, Houston, TX, USA).

### 2.5 Immunofluorescence

HL‐1 atrial myocytes were cultured on Millicell EZ slide 8‐well (Millipore). HL‐1 cells were treated with TWEAK for 24 hours after transfection of siRNA Fn14. After treatment, cells were washed with PBS and then fixed with 4% paraformaldehyde for 20 minutes at room temperature. Cells were washed thrice with PBS, blocked in 5% bovine serum albumin buffer for 1 hour and then were incubated overnight at 4°C with the primary antibodies against Fn14 (1:100 dilution; OmnimAbs). Cells were gently washed thrice with PBS before incubation with FITC‐conjugated secondary antibody (1:200 dilution; Bioss, Beijing, China) for 30 minutes at 37°C. Nuclei were stained with DAPI (Abcam, Cambridge, UK).

### 2.6 Enzyme‐linked immunosorbent assay

Human serum samples were collected from the same patients as isolation of PBMCs and stored at −80°C, and levels of TWEAK were determined using enzyme‐linked immunosorbent assay (ELISA) kits (eBioscience, Vienna, Austria) following the manufacturer's protocols. The sensitivity for the assay was 9.7 pg/mL. The intra‐assay and inter‐assay coefficient of variation was 7.9% and 9.2%, respectively.

### 2.7 Statistical analysis

All statistical analyses were performed using SPSS 17.0 (SPSS, Chicago, IL, USA). Continuous variables are represented as means ± SD and were compared using unpaired t test and one‐way ANOVA. Categorical variables were presented as counts and percentages then analysed using either a chi‐square test or Fisher's exact test. Logistic regression analysis was used to identify independent predictors of AF. A difference with a *P* value of <.05 (2‐sided) was considered statistically significant.

## 3 RESULTS

### 3.1 Lower TWEAK serum levels and up‐regulated TWEAK expression in PBMCs from patients with AF

**Figure 1:**
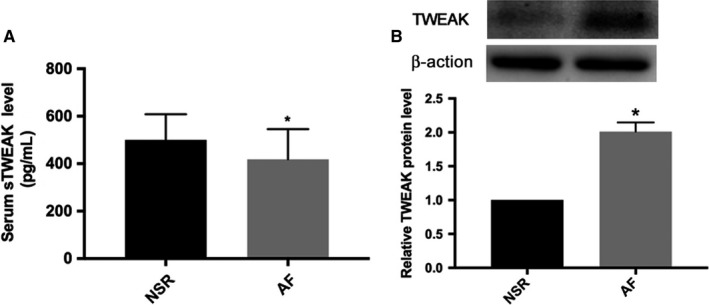
Decreased TWEAK serum levels and increased TWEAK expression in PBMCs from patients with AF. A, ELISA assay of serum TWEAK protein levels from NSR and AF subjects. B, Western blot analysis of TWEAK protein expression in PBMCs from NSR and AF subjects. AF, atrial fibrillation. NSR, normal sinus rhythm. PBMCs, peripheral blood mononuclear cells. *P <* *.05 vs NSR subjects group

To determine whether AF affected TWEAK expression, we examined the serum levels of TWEAK in AF and NSR subjects. White blood cells count and left atrial size were significantly higher in patients with AF than those in patients with NSR (6.35 ± 1.41 × 10^12^/L vs 5.61 ± 1.26 × 10^12^/L, *P *<* *.05 and 40.31 ± 5.08 mm vs 36.10 ± 4.84 mm, *P *<* *.05, respectively). There were no other significant differences between the two groups. The characteristics of the studied population were showed in Table 1. ELISA assay revealed significantly lower TWEAK serum levels in the AF group than NSR group (418.56 ± 127.28 pg/mL vs 500.5 ± 107.67 pg/mL, *P *<* *.05; Figure 1A). We have analysed the possible biomarkers of AF by univariate and multivariate regression analysis and found serum TWEAK level was an independent biomarker of AF (Table S1). To further confirm the effects of AF on TWEAK expression, we isolated PBMCs from blood samples. TWEAK expression was significantly higher in PBMCs from AF subjects as compared to NSR subjects (*P *<* *.05; Figure 1B).

**Table 1:** Characteristics of the patients

Variables	Control = 20	AF = 42	*P* value
Age (y)	55.25 ± 8.42	57.76 ± 10.14	.34
Male (%)	10 (50)	25 (60)	.48
Body mass index (kg/m^2^)	25.13 ± 3.29	26.82 ± 3.65	.09
Systolic pressure (mm Hg)	133.25 ± 21.72	132.33 ± 18.55	.86
Diastolic pressure (mm Hg)	80.25 ± 12.91	81.19 ± 12.28	.78
Medical history			
Hypertension (%)	8 (40)	24 (57)	.21
Diabetes (%)	2 (10)	9 (21)	.46
Coronary artery disease (%)	1 (5)	7 (17)	.38
Tobacco Abuse (%)	5 (25)	12 (29)	.77
Alcohol Abuse (%)	4 (20)	11 (26)	.83
Concomitant medications			
ACEI or ARB (%)	5 (25)	16 (38)	.31
Beta blocker (%)	9 (45)	26 (62)	.21
Calcium channel blockers (%)	2 (10)	9 (21)	.46
White blood cells count (x 10^12^/L)	5.61 ± 1.26	6.35 ± 1.41	<.05
Alanine transaminase	22.85 ± 8.50	21.21 ± 8.54	.48
Triglyceride	1.27 ± 0.69	1.49 ± 0.79	.29
Low‐density lipoprotein cholesterol	2.37 ± 0.67	2.63 ± 0.86	.23
Serum urea nitrogen	4.41 ± 1.14	4.54 ± 1.41	.73
Serum creatine	60.95 ± 11.56	66.74 ± 13.84	.11
Left atrial size (mm)	36.10 ± 4.84	40.31 ± 5.08	<.05
Left ventricular ejection fraction	0.63 ± 0.05	0.61 ± 0.04	.14

ACEI, angiotensin‐converting enzyme inhibitors; ARB, angiotensin receptor blockers.

### 3.2 Increased Fn14 expression in atrial appendages of patients with AF

**Figure 2:**
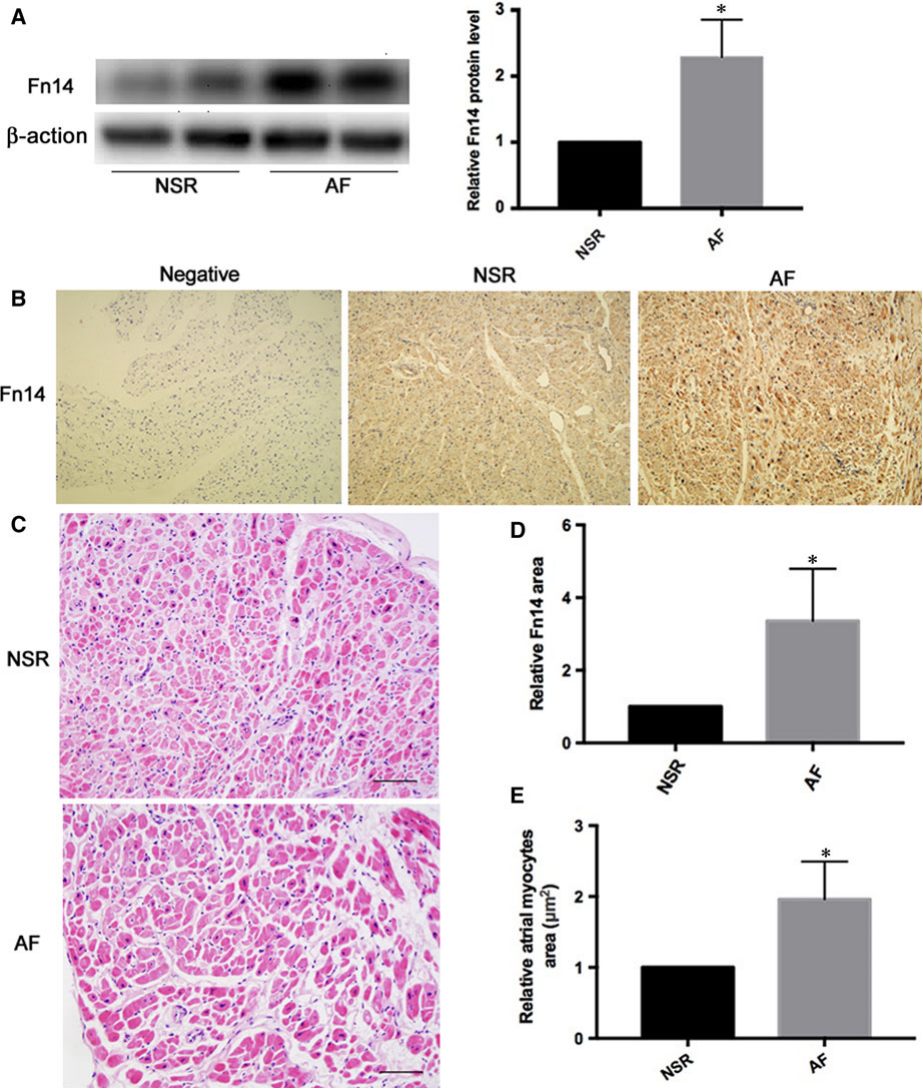
Tissue levels of Fn14 from atrial appendage were higher in patients with AF than those with NSR. A, Western blot analysis of Fn14 protein expression from NSR and AF subjects. B, Fn14 protein expression from NSR and AF subjects by immunohistochemistry. C, Representative HE staining of transverse sections from human atrial appendages (scale bar: 50 μm). D, Quantitative analysis of B. E, Quantitative analysis of C. AF, atrial fibrillation. NSR, normal sinus rhythm. *P <* *.05 vs NSR subjects group

To confirm the effects of AF on Fn14 expression, we used Western blot analysis and immunohistochemistry of atrial appendages from AF and NSR subjects. Fn14 protein expression was higher in atrial appendages from AF than NSR subjects by both Western blot analysis and immunohistochemistry (*P *<* *.05; Figure 2A,B).

### 3.3 H&E staining showed larger atrial myocytes area in atrial appendages from patients with AF

In H&E‐stained sections, atrial myocyte area (μm^2^) was increased in atrial appendages from AF patients (*P *<* *.05; Figure 2C,E).

### 3.4 TWEAK‐induced hypertrophy of HL‐1 atrial myocytes and increased Fn14 expression

**Figure 3:**
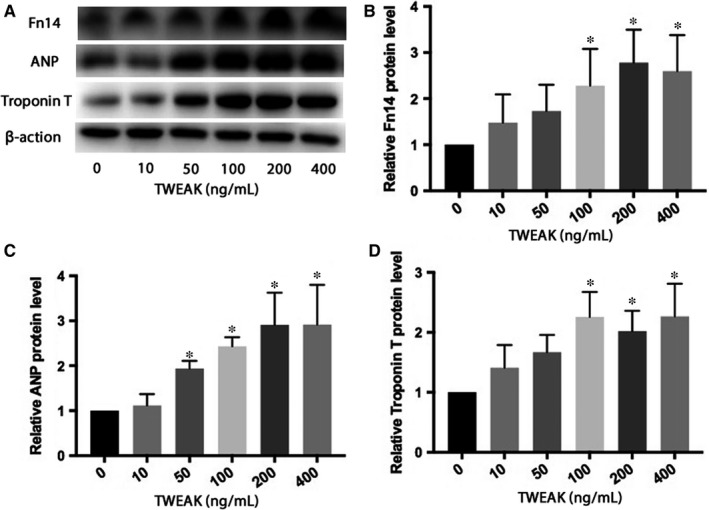
TWEAK increased the expression of Fn14 and induced hypertrophy in HL‐1 atrial myocytes. A, Western blot analysis of Fn14, ANP, Troponin T protein expression after TWEAK stimulation at different concentrations. B‐D, Quantitative analysis of A. *P <* *.05 compared with control

Based on previous studies, we chose a series of TWEAK concentrations to explore the effect of TWEAK on hypertrophy of HL‐1 atrial myocytes. Relative to NC treatment, TWEAK at 50, 100, 200 and 400 ng/mL increased ANP expression (*P *<* *.05; Figure 3A,C), and at the latter 3 concentration also Troponin T expression (*P *<* *.05; Figure 3A,D). Meanwhile, we evaluated the Fn14 expression in TWEAK‐treated HL‐1 atrial myocytes at different concentration. The Fn14 protein level increased at 100, 200 and 400 ng/mL with TWEAK (*P *<* *.05; Figure 3A,B). Thus, Fn14 expression was positively regulated by TWEAK in HL‐1 cells in vitro, and the concentration of 100 ng/mL as used as TWEAK stimulation in the following vitro experiments.

### 3.5 Fn14 inhibition attenuated the increased hypertrophy induced by TWEAK stimulation in HL‐1 atrial myocytes

**Figure 4:**
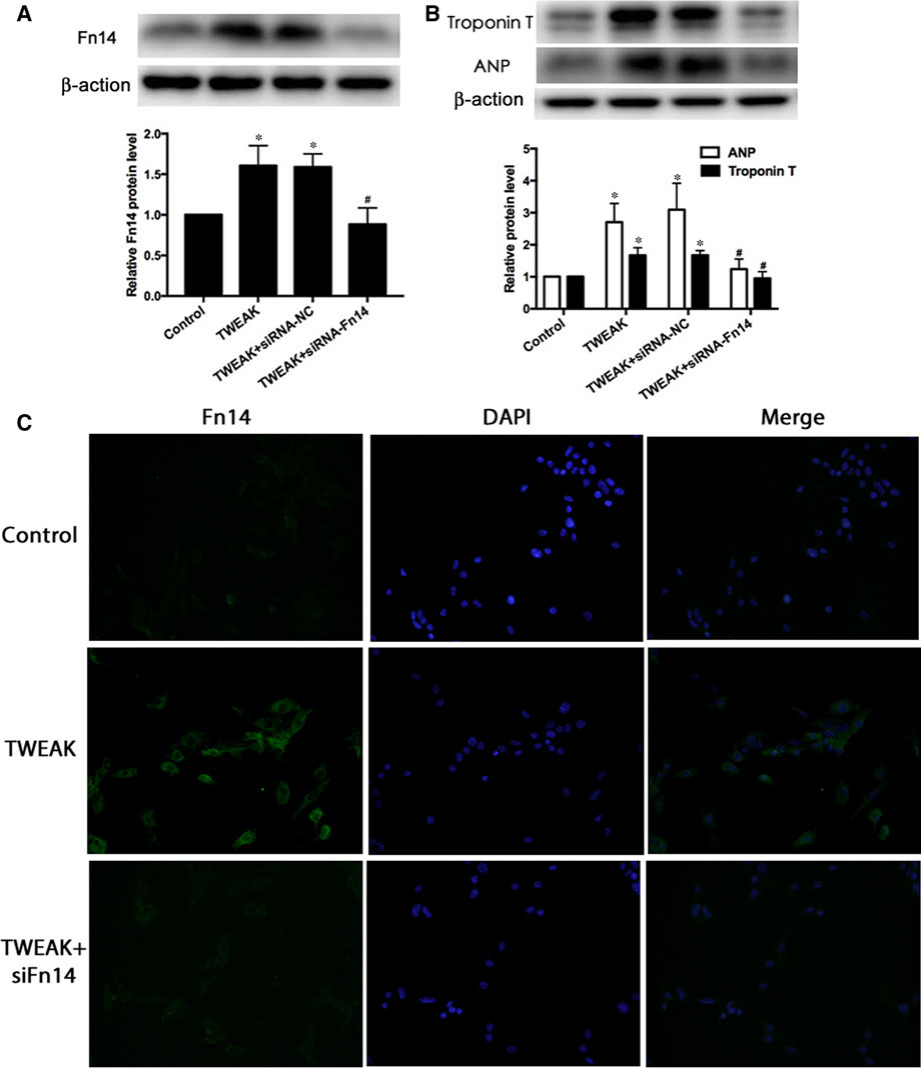
Fn14 knockdown ameliorated HL‐1 atrial myocytes hypertrophy induced by TWEAK stimulation. A, B Western blot analysis of Fn14, ANP, Troponin T protein expression in HL‐1 atrial myocytes with Fn14 siRNA knockdown. C, Immunofluorescence for Fn14 (green) and DAPI (blue) demonstrating increased Fn14 levels after TWEAK (100 ng/mL) treatment for 24 h; Fn14 siRNA inhibition attenuated the effect in HL‐1 atrial myocytes. *P <* *.05 vs control without treatment and ^#^ *P *<* *.05 vs only TWEAK

To determine the effect of Fn14 on atrial myocytes hypertrophy, we used Fn14‐specific siRNA to knock down Fn14 expression in HL‐1s. Fn14 expression significantly decreased after transfection with Fn14‐specific siRNA as compared to siNC treatment (*P *<* *.05; Figure 4A). Immunofluorescence assay also revealed greater Fn14 protein level with TWEAK stimulation than control conditions, and reduced Fn14 protein expression following Fn14 inhibition for 24 hours (*P *<* *.05; Figure 4C). TWEAK stimulation significantly increased HL‐1 atrial myocytes hypertrophy, while inhibition of Fn14 attenuated the increased ANP and Troponin T protein expression induced by TWEAK (*P *<* *.05; Figure 4B). Therefore, TWEAK‐induced HL‐1 atrial myocytes hypertrophy was reduced following the inhibition of Fn14, rendering Fn14 responsible for TWEAK‐induced HL‐1s hypertrophy.

### 3.6 Activation of JAK2/STAT3 pathway by TWEAK/Fn14 in HL‐1 atrial myocytes

**Figure 5:**
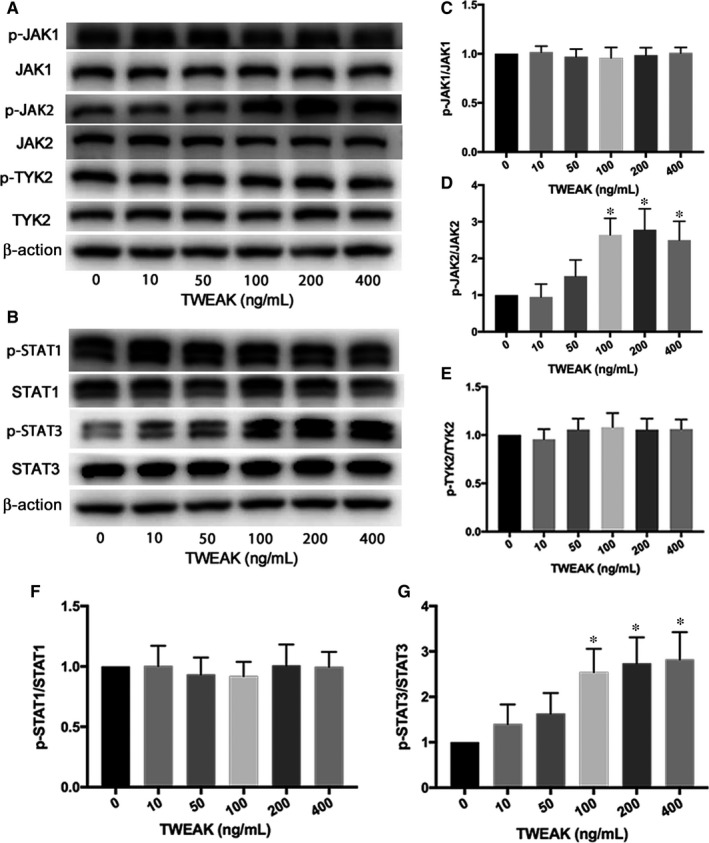
TWEAK increased p‐JAK2 and p‐STAT3 expression in HL‐1 atrial myocytes. A, B, Western blot analysis of p‐JAK1, p‐JAK2, p‐TYK2 and p‐STAT1, p‐STAT3 protein expression after TWEAK stimulation at different concentrations. C‐G, Quantitative analysis of A, B. *P <* *.05 compared with control

**Figure 6:**
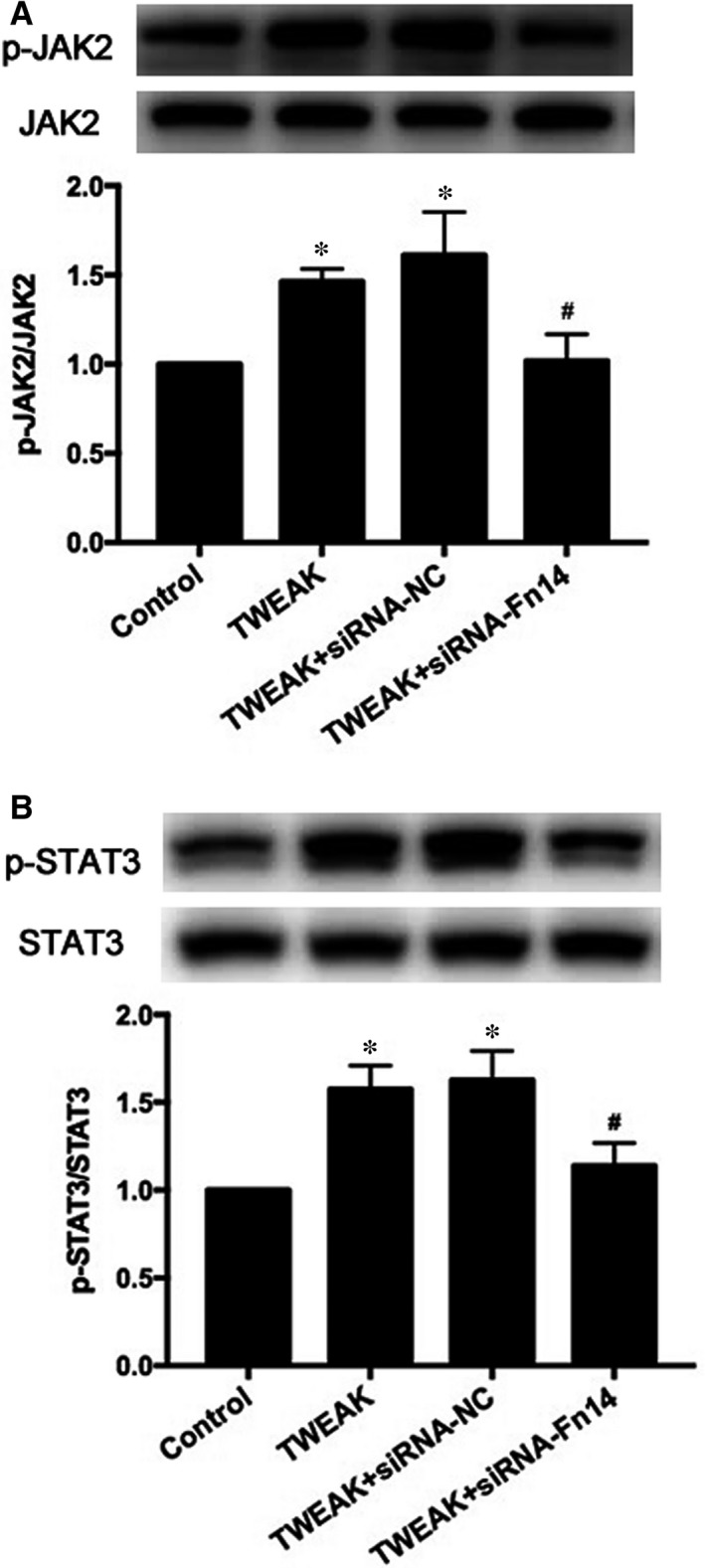
Inhibition of Fn14 effectively decreased TWEAK‐induced p‐JAK2 and p‐STAT3 expression. A, Western blot analysis of p‐JAK2 and JAK2 protein expression after treatment with Fn14 siRNA. B, Western blot analysis of p‐STAT3 and STAT3 protein expression after Fn14 knockdown by siRNA. *P <* *.05 vs control without treatment and ^#^ *P *<* *.05 vs only TWEAK

To assess whether JAK/STAT pathway was associated with TWEAK/Fn14 activation in HL‐1 atrial myocytes, we explored JAK/STAT expression with TWEAK treatment. p‐JAK2 and p‐STAT3 expression significantly increased after TWEAK treatment for 24 hours (*P *<* *.05; Figure 5A,B,D,G). p‐JAK1, p‐TYK2, p‐STAT1 protein expression were also evaluated, and no obvious changes were observed after TWEAK stimulation (Figure 5A‐C,E,F). In addition, Western blot analysis showed that inhibition of Fn14 effectively decreased TWEAK‐induced p‐JAK2 and p‐STAT3 expression (*P *<* *.05; Figure 6A,B).

### 3.7 Fn14 mediated TWEAK‐induced hypertrophy via JAK2/STAT3 pathway in HL‐1 atrial myocytes

**Figure 7:**
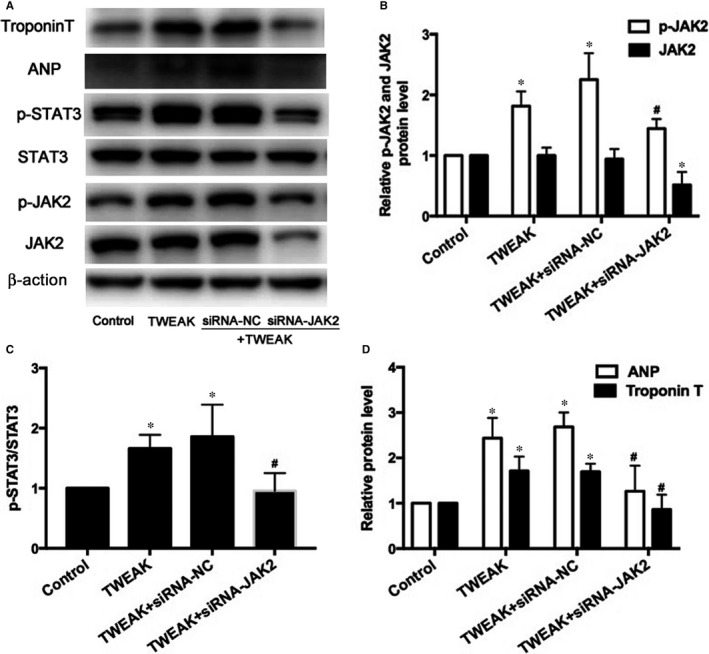
Effects of JAK2 knockdown by siRNA on the expression of phosphorylation of STAT3 and atrial hypertrophy in HL‐1 myocytes. A, Protein expression of p‐JAK2, JAK2, p‐STAT3, STAT3, ANP and Troponin T after treatment with JAK2 siRNA. B, C, D, Quantitative analysis of A. *P <* *.05 vs control without treatment and ^#^ *P *<* *.05 vs only TWEAK

**Figure 8:**
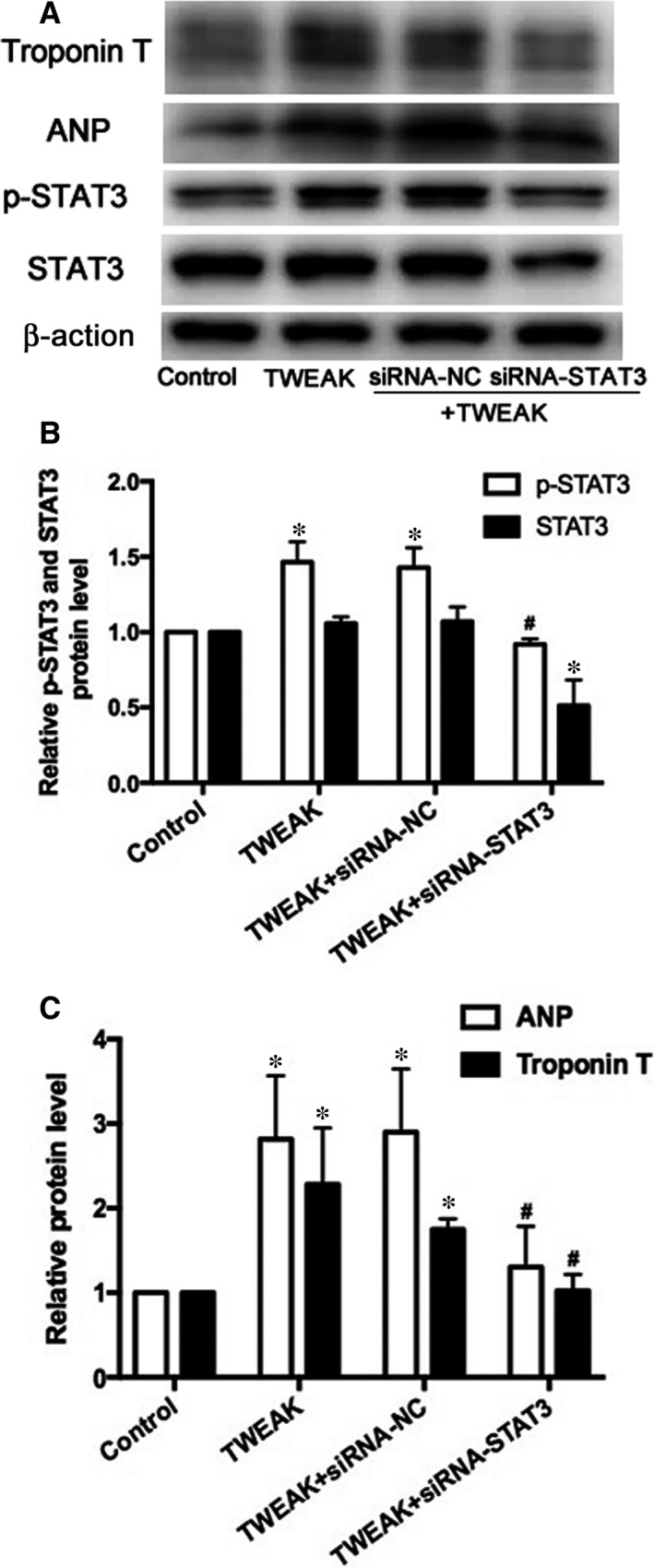
Effects of STAT3 interference by siRNA on the expression of atrial hypertrophy in HL‐1 myocytes. A, Protein expression of p‐STAT3, STAT3, ANP and Troponin T after treatment with STAT3 siRNA. B, C, Quantitative analysis of A. *P <* *.05 vs control without treatment and ^#^ *P *<* *.05 vs only TWEAK

Because JAK2/STAT3 pathway can be modulated by TWEAK/Fn14 axis, we explored the potential role of the JAK2/STAT3 pathway in TWEAK‐modulated hypertrophy. Following the blockade of JAK2 expression by siRNA (*P *<* *.05; Figure 7A,B), p‐STAT3, ANP and Troponin T protein levels decreased significantly (*P* < .05; Figure 7A,C,D). Similarly, siRNA knockdown of STAT3 (*P *<* *.05; Figure 8A,B) decreased ANP and Troponin T expression (*P *<* *.05; Figure 8A,C). Therefore, TWEAK up‐regulated ANP and Troponin T expression by activating the JAK2/STAT3 pathway.

## 4 DISCUSSION

Atrial myocyte hypertrophy can lead to conduction heterogeneity and is one of the most important substrates in AF.^16^, ^17^ TWEAK/Fn14 axis is a positive regulator of cardiac hypertrophy in cardiomyopathy.^6^, ^9^ The present study focused on the potential role and mechanism of Fn14 in TWEAK‐induced atrial myocyte hypertrophy. The major findings were that in patients with AF, Fn14 protein levels were increased in atrial myocytes and TWEAK expression was up‐regulated in PMBCs while TWEAK serum levels were decreased; in vitro, TWEAK increased Fn14 expression, and Fn14 siRNA knockdown counteracted the increased hypertrophy induced by TWEAK; activation of JAK2/STAT3 pathway was critical to the TWEAK‐induced hypertrophy in HL‐1 atrial myocytes. Collectively, this study firstly proved that TWEAK/Fn14 axis contributed to HL‐1 atrial myocyte hypertrophy via JAK2/STAT3 pathway. However, further detailed investigations are needed to clarify the molecular mechanism by which TWEAK/Fn14 modulates atrial myocytes hypertrophy in vivo.

Atrial fibrillation is clearly associated with increased levels of known inflammatory markers, and inflammation plays a role in the genesis and perpetuation of AF.^18^, ^19^, ^20^ TWEAK is a member of the tumour necrosis factor (TNF) pro‐inflammatory cytokine superfamily.^3^, ^21^ Consistent with leucocytes as a main cellular source of TWEAK, TWEAK is expressed broadly by human peripheral blood‐derived innate immune cells.^22^, ^23^ Thus,we examined the expression of TWEAK in PBMCs and found that TWEAK levels were increased in PBMCs from patients with AF. Increased peripheral blood leucocytes count has been observed in AF group^24^, ^25^ which is a main source of TWEAK.^22^, ^23^ Besides, PBMCs may produce TWEAK in an incompletely compensatory manner to counter the decreased serum levels of TWEAK.

TWEAK is expressed as a type II transmembrane protein that can be processed to generate a soluble cytokine.^3^ Elevated circulating levels of TWEAK are sufficient to cause cardiac dilation and progression to heart failure in adult TWEAK transgenic overexpression mice.^6^ However, it has been reported that decreased concentrations of sTWEAK have been observed in diseases related to chronic low‐grade inflammation,^26^, ^27^ and TWEAK may have evolved to guard against the development of a potentially excessive inflammatory response.^22^, ^26^ Previous studies have demonstrated that subjects with carotid stenosis showed a reduced plasma sTWEAK level compared with healthy subjects, and sTWEAK concentrations negatively correlated with the carotid intima‐media thickness.^26^ Decreased sTWEAK concentration is significantly and independently associated with long‐term cardiovascular mortality in patients with peripheral arterial disease.^27^ TWEAK blood plasma levels are reduced in monocrotaline‐treated rats while Fn14 is up‐regulated in the heart.^5^ Consistent with previous studies, we found that the levels of serum TWEAK in patients with AF were significantly lower than those of NSR controls. The mechanisms leading to decreased serum levels of sTWEAK are not clear. Circulating levels of sTWEAK may be influenced by the proportion of sTWEAK bound to its scavenger receptor CD163 and its receptor Fn14. On the one hand, it might be related to scavenging capacity of CD163 which was exclusively expressed by monocytes and macrophages and the sTWEAK down‐regulation might result from increased concentration of serum CD163 as a compensatory mechanism to protect from the consequences of TWEAK/Fn14 activation.^5^, ^28^ On the other hand, sTWEAK may exert some biological effects on a variety of tissues by binding to Fn14 and reduced sTWEAK serum levels might be because of the up‐regulated Fn14 expression.^5^ Additionally, sTWEAK might be excreted more and decrease because of diuretic effect of increased ANP secretion with the onset of atrial fibrillation.^29^, ^30^, ^31^

Some recent studies have focused on the role of TWEAK in the heart. TWEAK is a positive regulator of proliferation in neonatal but not adult rat cardiomyocytes because of developmental down‐regulation of its receptor Fn14.^4^ TWEAK stimulation results in adult rat cardiomyocyte hypertrophy,^9^ and transgenic mice overexpressing TWEAK exhibit cardiomyocyte hypertrophy.^6^ TWEAK mediates the processes mainly through the Fn14 receptor via various downstream signalling pathways.^4^, ^5^ TWEAK itself can induce Fn14 expression when added to glioma cell lines.^32^ Consistent with previous research, we found that Fn14 was highly induced by TWEAK stimulation in HL‐1 atrial myocytes. Cardiomyocytes hypertrophy is characterized by an increased expression of hypertrophic marker such as ANP and Troponin T.^17^, ^33^ Our findings also demonstrated that TWEAK increased ANP and Troponin T expression in HL‐1 atrial myocytes, and inhibition of Fn14 attenuated said induced expression. Therefore, Fn14 was required for TWEAK‐induced HL‐1 atrial myocytes hypertrophy. In addition, we found that the expression of Fn14 protein was elevated in atrial appendages from patients with AF. These results suggest that inhibition of Fn14 may protect atrial myocytes against hypertrophy and even prevent the occurrence and progression of AF.

We further investigated the potential mechanism of TWEAK‐induced HL‐1 atrial myocytes hypertrophy in vitro. Fn14 associates with TNF receptor‐associated factors and mainly signal through NF‐κB pathways.^21^, ^34^ However, TWEAK‐induced cardiac hypertrophy in mice is independent of TNF signals, and TWEAK activates nuclear translocation of p65 as well as p50 in isolated adult cardiomyocytes.^6^ Another important mediator is the JAK/STAT pathway, which is involved in vascular atherosclerosis and ventricular hypertrophy.^35^, ^36^ TWEAK/Fn14 mediates tumour cell apoptosis through JAK‐STAT pathway.^37^ The phospho‐STAT3 levels are elevated in human atrial tissues from patients with AF, and angiotensin II (Ang II)‐induced JAK/STAT activation in a rat model localizes mainly to the atrium.^1^ In hepatic stellate cells, TWEAK up‐regulated the expression of Fn14 and pro‐inflammatory cytokine secretion through NF‐κB/STAT3 pathways.^13^ In the present study, activation of Fn14 up‐regulated both p‐JAK and p‐STAT3 level in HL‐1 atrial myocytes, while in vitro knockdown of Fn14 attenuated the increased expression of p‐JAK and p‐STAT3.

The JAK‐STAT pathway plays an important role in cardiomyocyte cytokine signalling. Mechanical stretch‐induced activation of matrix metalloproteinases in neonatal rat cardiomyocytes is mediated by Ang II‐JAK‐STAT pathway.^38^ JAK‐STAT signalling has been implicated in pressure overload‐induced cardiac hypertrophy and remodelling.^12^ Ang II promotes the activation of JAK/STAT and expression of ANP in atrial myocytes.^1^ In the present study, in vitro knockdown of JAK2 with specific siRNA attenuated the elevated expression of p‐STAT3, ANP and Troponin T induced by TWEAK. Inhibiting STAT3 reduced the TWEAK‐induced expression of ANP and Troponin T. These findings underscored the roles of the JAK2/STAT3 pathway in TWEAK‐induced atrial myocytes hypertrophy, and suggested that TWEAK/Fn14 axis promoted atrial myocytes hypertrophy via the activation of JAK2/STAT3 pathway.

The present study included some limitations. The major limitation of the study is that there is no AF animal model for validation in vivo. The number of human samples was limited, although statistical significance had been reached. Another limitation is that the serum levels of scavenger receptor CD163 were not detected.

In conclusion, we provide strong evidence that TWEAK induces Fn14 expression, which subsequently contributes to atrial myocytes hypertrophy. Additionally, JAK2/STAT3 pathways play an essential role in TWEAK‐induced atrial myocytes hypertrophy. This study provides a better understanding of the regulatory mechanisms of TWEAK/Fn14 axis in atrial hypertrophy. Fn14 gene silencing may protect against atrial hypertrophy and may be a useful approach in treatment of atrial structural remodelling.

This study was approved by the local institutional review board and conducted according to the Declaration of Helsinki. Signed informed consent was provided by each patient involved.

## CONFLICTS OF INTEREST

The authors confirm that there are no conflicts of interest.
