# Supplementary material for: TWEAK/Fn14 mediates atrial‐derived HL‐1 myocytes hypertrophy via JAK2/STAT3 signalling pathway
Source: J Cell Mol Med. 2018 Jul 4;22(9):4344–53. doi: 10.1111/jcmm.13724 (PMC6111870; doi:10.1111/jcmm.13724)
Supplement: Supplementary file 1 [file JCMM-22-4344-s001.doc]

| **Table S1. Univariate and multivariate predictors of AF.** | | | | | | |  | |
| --- | --- | --- | --- | --- | --- | --- | --- | --- |
| Variables | Univariate Analysis | | |  | Multivariate Analysis | | | |
|  | OR 95%CI p-value | | |  | OR 95%CI p-value | | | |
| Age | 2.333 | 0.753-7.235 | 0.142 | |  |  | |  |
| Body mass index | 2.000 | 0.675-5.926 | 0.211 | |  |  | |  |
| Hypertension | 2.000 | 0.677-5.909 | 0.210 | |  |  | |  |
| White blood cells count | 3.343 | 1.096-10.192 | 0.034 | |  |  | |  |
| Left atrial size | 4.000 | 1.153-13.876 | 0.029 | |  |  | |  |
| Serum TWEAK | 0.269 | 0.088-0.826 | 0.022 | | 0.269 | 0.088-0.826 | | 0.022 |

AF, atrial fibrillation; CI, confidence OR, odd ratio.
